# Supplementary material for: FastqPuri: high-performance preprocessing of RNA-seq data
Source: BMC Bioinformatics. 2019 May 3;20:226. doi: 10.1186/s12859-019-2799-0 (PMC6500068; doi:10.1186/s12859-019-2799-0)
Supplement: Supplementary file 2 — Archive of FastqPuri. Archive containing all files needed to install and run FastqPuri v1.0.6. Date stamp March 22, 2019. (GZ 47,819 kb) [file 12859_2019_2799_MOESM2_ESM.gz › FastqPuri-1.0.6/html/struct__ds__adap.html]

FastqPuri: \_ds\_adap Struct Reference


FastqPuri

Public Attributes |
List of all members

\_ds\_adap Struct Reference

structure containing an adapter pair (for read 1 and read 2)
More...

`#include <trimDS.h>`

|  |  |
| --- | --- |
| Public Attributes | |
| char | ad1 [READ\_MAXLEN] |
|  | |
| char | ad2 [READ\_MAXLEN] |
|  | |
| int | L1 |
|  | |
| int | L2 |
|  | |

## Detailed Description

structure containing an adapter pair (for read 1 and read 2)

## Member Data Documentation

## ◆ ad1

char \_ds\_adap::ad1[READ\_MAXLEN]

read 1 associated adapter sequence

## ◆ ad2

char \_ds\_adap::ad2[READ\_MAXLEN]

read 2 associated adapter sequence

## ◆ L1

int \_ds\_adap::L1

adapter 1 sequence length

## ◆ L2

int \_ds\_adap::L2

adapter 2 sequence length

---

The documentation for this struct was generated from the following file:

- include/trimDS.h


---

Generated on Mon Mar 19 2018 23:42:01 for FastqPuri by  

 1.8.14
